# Forces influencing generic drug development in the United States: a narrative review

**DOI:** 10.1186/s40545-016-0079-1

**Published:** 2016-09-22

**Authors:** Chia-Ying Lee, Xiaohan Chen, Robert J. Romanelli, Jodi B. Segal

**Affiliations:** 1 Johns Hopkins University Bloomberg School of Public Health, Center for Drug Safety and Effectiveness, 624 N. Broadway, Room 644, Baltimore, MD 21205 USA; 2 Palo Alto Medical Foundation Research Institute, Palo Alto, CA USA; 3 Division of General Internal Medicine, Johns Hopkins University School of Medicine, 624 N. Broadway, Room 644, Baltimore, MD 21205 USA

**Keywords:** Generic drug, Incentives, U.S. Food and Drug Administration

## Abstract

**Background:**

The United States (U.S.) Food and Drug Administration, as protectors of public health, encourages generic drug development and use so that patients can access affordable medications. The FDA, however, has limited mechanisms to encourage generic drug manufacturing.

**Main results:**

Generic drug manufacturers make decisions regarding development of products based on expected profitability, influenced by market forces, features of the reference listed drug, and manufacturing capabilities, as well as regulatory restrictions. Barriers to the development of generic drugs include the challenge of demonstrating bioequivalence of some products, particularly those that are considered to be complex generics.

**Conclusions:**

We present here a focused review describing the influences on generic manufacturers who are prioritizing drugs for generic development. We also review proposed strategies that regulators may use to incentivize generic drug development.

## Background

The use of generic drugs products typically yields lower costs to insurers and patients than use of branded products. Efforts to increase generic drug utilization have proven helpful in reducing healthcare spending in the United States (U.S.) [1]. According to the most recent report from the Generic Pharmaceutical Association, generic drugs accounted for 88 % of all dispensed retail prescriptions in the U.S in 2014, while consuming only 28 % of total drug spending [2]. The use of generics, where available, is estimated to have saved the U.S. healthcare system $1.68 trillion between 2005 and 2014, with $254 billion saved in 2014 alone [2]. Patent expiration for a number of blockbuster drugs from 2010 to 2014, followed by the launch of generic equivalents, has played an important role in healthcare cost savings.

Despite evidence that generic drugs bring value to the healthcare system, these products are not uniformly valuable to their manufacturers. Manufacturers are appropriately deliberate about investing in the development of generic products. The United States (U.S.) Food and Drug Administration, as protectors of public health, encourages generic drug development and use so that patients can access affordable medications; however, the FDA has few mechanisms to incentivize generic manufacturing. Herein, we present a review aimed at exploring the forces that limit the development and production of generics in the U.S. and the ways in which the FDA could conceivably reduce barriers to the development of generic drugs. Our objective was to review the influences on manufacturers, which are the parties responsible for prioritizing drugs for generic development, as well as the strategies that regulators may use to incentivize generic drug development in the U.S.

### Pathways to generic drug development

The Drug Price Competition and Patent Term Restoration Act of 1984, often referred to as the Hatch-Waxman Act, amended the Federal Food, Drug, and Cosmetic Act to create an abbreviated pathway for the approval of new drugs that are therapeutically equivalent to a branded drug [1]. Under this Act, a manufacturer needs only to demonstrate the bioequivalence of a generic product to a reference listed drug through an abbreviated new drug application (ANDA), rather than repeating the costly and time consuming safety and efficacy studies required of innovative, new chemical entities [3–5]. The FDA considers a generic drug to be bioequivalent to the reference listed drug if the rate and extent of absorption of the generic drug do not show a significant difference from the rate and extent of absorption of the listed drug when administered at the same molar dose of the therapeutic ingredient under similar experimental conditions in either a single dose or multiple doses [3]. Pharmaceutically equivalent drugs are products with the same active ingredients, dosage form, strength, and route of administration. Of these, those proven to be *bioequivalent* are consequently considered therapeutically equivalent or substitutable - with the expectation that they have the same safety and efficacy profile. Once the FDA approves the ANDA and the branded version is no longer protected by patent or market exclusivity, the generic product can be brought to market.

When identifying a new development target, generic manufacturers first seek an informed perspective on the competitive landscape about potential generic competitors before prioritizing resources and making a decision to proceed [6]. Strategies for generics development have evolved rapidly, responding to market dynamics [7]. Between 1984 and 2012, generic manufacturers focused largely on the production of “simple” to formulate small molecules, and won the market via mass production. The years between 2012 and 2017 have been called the “patent cliff”, when many drugs considered to have been “blockbuster drugs’ lost, or will soon lose, their patent protection. This opened important opportunities for generic manufacturers, who commonly apply one or more strategies. The first is the “portfolio-centric approach”. Generic manufacturers include re-innovation design (i.e., the process of producing the next generation of generics with revised and refined features of successfully-launched products) in their portfolio ostensibly to provide personalized, cost-effective generic products that meet the demand of healthcare systems, policymakers, and patients [8]. One way is through what is called re-innovation or sometimes called the production of “super generics”. The products have modifications beyond the originator generic; these often require a submission to FDA of a new drug application rather than an ANDA. An example is the generic drug, abraxane, which the manufacturer expected to gain market share over paclitaxel. The active ingredient, paclitaxel, is unchanged but abraxane has the molecule coated in albumin, allowing the company to claim fewer adverse reactions for the patient [9, 10]. The second is the “therapeutic area dominance”. With this approach, generic manufacturers compete within a specialized area of generic drugs such as cardiovascular, oncology, or rheumatology drugs [11]. For example, Relax Pharmaceuticals (an Indian generic manufacture) specializes in the manufacturing of generic antibiotics and gastrointestinal products. Finally, market experts have predicted that after 2017, when many branded drugs have lost their patent protection, generic manufacturers will shift resources to the production of complex generics such as drug/device combinations and sterile injectables, or may move into the marketplace for biosimilar products [5, 12].

### Manufacturing considerations

When generic manufacturers are selecting products for development, the expectation of an adequate return on investment is the foremost consideration. This can be forecast, to some extent, by knowing the demand for the product and the requirements for production.

#### Market forces

Generic manufacturers understandably focus on drugs with a potential for high profit. Companies are often interested in developing a generic if the branded medication has a high average wholesale price, which is expected to translate into a profitable generic. Similarly, chronic diseases, with a larger population health burden, are also often targeted areas for generic manufacturers. For example, cardiovascular and central nervous system diseases are the two largest market segments, composing nearly 38 % of the global generic pharmaceutical market together [13, 14]. Generic manufacturers may also choose to avoid densely populated therapeutic classes, when deciding whether to join the competition.

#### Features of reference listed drug

The Hatch-Waxman Act defined single-dose pharmacokinetic studies as the method of establishing bioequivalence for *systemically* acting drug products. However, there are formulations or routes of administration where systemic blood levels cannot be used to determine bioequivalence, such as when the product is not intended to be systemically active. Therefore, drugs that are used topically in the eye; or dosage forms intended to act within the gastrointestinal tract lumen without absorption, do not have clear paths for demonstrating bioequivalence, which is a major deterrent to their production. The FDA has made significant progress in defining bioequivalence methodologies for many of these non-systemically active drug products; [15] however, a number of products remain without clearly defined methodology, including inhaled drug products and transdermal preparations. This impacts the production of generics for inhaled corticosteroids, such as for asthma treatment, and transdermal testosterone for hormone replacement, as examples. In the absence of product-specific guidance, drug developers may ask the FDA development questions via the controlled correspondence process or request to meet with FDA scientists in pre-ANDA meetings. Sponsors also may develop their own analytical tools and study protocols through which they can demonstrate bioequivalence, and then work with the FDA for approval of their plan [2].

#### Manufacturing capabilities of complex drugs

Generic manufacturers also consider their technical ability to manufacture generic versions of branded products. They evaluate the complexity of the manufacturing that is needed to produce a generic equivalent, the cost of manufacturing, and their own research and development capabilities. For example, small molecule pharmaceutical products are simpler to create and therefore are more likely to be developed generically than complex large molecules. Dosage form also may play a role in generic drug development; solid oral forms (e.g., tablets, capsules) and parenteral solutions have a higher likelihood of being easily developed than more complex drug delivery systems (again, inhaled products, topical products). These differences are due to technological barriers and manufacturers’ capabilities. For example, the drug delivery system development process is particularly complicated for inhaled products with manufacturers needing to work around the bans on ozone-depleting propellants and the multiple patents on hydrofluorocarbon-free devices. Generic manufacturers need sufficient research and development resources to manufacture these technically challenging generic products.

Innovative products have become increasingly complex and, necessarily, so have their generic equivalents [16]. Each complex generic is “complex” in its own way [17, 18] (Table 1). The development of complex generics requires substantial commitment from the manufacturer, which may lower the benefit to cost ratio for its production. The manufacturer may be asked to repeat clinical studies due to the challenges of demonstrating bioequivalence for these products or do additional physiochemical characterization testing [11]. Furthermore, for complex generics the pathway to drug approval may differ substantially from one product to another product; [13] the FDA’s Office of Generic Drugs (OGD) manages each submission on a case-by-case basis. Complex generics are expected to eventually become a significant percentage of the generic market, but the approval challenges first must be overcome.

**Table 1:** Complex generics examples [11]

Complex Active Ingredients	Peptides, complex mixtures, natural source products
Complex Formulations	Liposomes and iron colloids
Complex Route of Delivery	Locally acting drugs
Complex Drug-Device Combinations	Metered dose inhaled products and transdermal systems

#### Materials challenges

Some generic products are challenging to develop because of inadequate source raw materials, although this is not unique to generics. The International Conference on Harmonization of Technical Requirements for Registration of Pharmaceuticals for Human Use guidelines are the main instructions for sourcing qualified raw materials for any drug manufacturing [19]. Drugs with carcinogenicity or toxicities may require suitable handling conditions in clinical practice and during development, which may be a deterrent to production, particularly if the return on investment cannot be assured [20, 21].

### Regulatory considerations

#### 180-day exclusivity period

The Hatch-Waxman Act gave additional protection to the inventors of innovative, new drugs by lengthening patent terms and by providing guaranteed periods of data exclusivity (5 years for a new chemical entity). The Act, in return, offers the first generic manufacturer to file an ANDA a 180-day period of market exclusivity. An effect of this regulation, however, is that the exclusivity period prevents other generic manufacturers from bringing their generic product to market during this time. This is typically not advantageous for consumers because it is not until roughly six generic products are available that the full cost savings of generics are typically realized; although the largest price decrease occurs with the entry of the second product [22].

#### Pay for delay

The 180-day exclusivity advantage provided to generic manufactures under the Hatch-Waxman Act encourages generic manufacturers to challenge the existing patents of brand manufacturers (“the Paragraph IV challenges”). It has, however, also encouraged generic drug manufacturers to settle and accept compensations from brand manufacturers for their delaying entry into the market [16]. This is counter to forces that encourage generic development and introduction to the market. Kesselheim describes studies measuring the impact of the Hatch-Waxman act; he notes that there are no well-controlled studies of the economic impact of Paragraph IV challenges and the effect of settlements on generic drug availability and public health outcomes [23].

These settlements have taken the form of a cash payment (so called “pay for delay”) to reimburse some or all of a generic manufacturer’s legal fees, or non-cash deals where the brand manufacturer agrees to purchase their product’s active ingredients from the generic manufacturer or not to market their own generic version of the product (i.e., an authorized generic) for a period of time [21]. In one notable case of cash payments, Cephalon made reverse payments totaling $300 million dollars to four generic drug manufacturers to drop patent challenges and suspend marketing of generic versions of Cephalon’s drug Provigil for six years. During that time, Cephalon earned an additional $4 billion dollars in sales for Provigil [24]. This was later found to be unlawful by the Federal Trade Commission (FTC) on the basis of these settlements being anticompetitive.

Pay for delay has posed a threat to the timely development of generic drugs and a substantial cost to the U.S. healthcare system. According to an FTC study, these deals cost consumers and taxpayers $3.5 billion in higher drug costs each year [25]. In 2013, there were more than 100 settlements reached between brand and generic manufacturers. In a suit filed by the FTC against the pharmaceutical company, Actavis, the U.S. Supreme Court ruled in 2013 that reverse payments are subject to U.S. antitrust laws [26]. Larger penalties to brand manufacturers, as well as generic manufacturers who accept such payments, may be needed to dissuade settlements that delay the availability of generic products. Hemphill and Lemley described a strategy called *earning exclusivity* that they proposed would improve the effect of the Hatch-Waxman Act on encouraging generic drug development [27]. They suggested that under the strategy of earning exclusivity, generic manufacturers would have to *earn* the 180-day exclusivity offered by FDA by successfully defeating the weak or bad patents on the branded products, without settlements. This would be done by demonstrating that the patent was invalid or by demonstrating that the generic product does not infringe upon the existing patent. Barr won an exclusive marketing rights challenge against Lilly for the production of a generic version of Prozac in 2000, which was 2 years before the patent was expected to expire [28].

#### Access to the branded reference products

Manufacturers, necessarily, must have access to the reference listed reference drug to perform the bioequivalence testing that the FDA requires of a generic product under an ANDA [29]. There have been instances, however, of brand manufacturers preventing generic manufacturers from accessing their product [23]. Some brand manufacturers have used the restrictions in their Risk Evaluation and Mitigation Strategies (REMS), and other restricted access programs, to prevent generic manufacturers from accessing brand drug samples for testing. These manufacturers have argued that they cannot provide generic manufacturers with samples of such REMS-covered products because doing so would be outside the FDA-sanctioned, restricted distribution pathway [30].

The Fair Access for Safe and Timely (FAST) Generics Act, first proposed in Congress in September 2014, was considered a solution to this REMS barrier [31]. The FAST Generics Act would create a pathway for generic manufacturers to secure a branded drug from its maker, wholesaler, or specialty distributor regardless of whether the product was subject to REMS, and impose stiff penalties for non-compliance. Ultimately, the branded drugs and their generic equivalents would then share a single REMS. At the time of writing, neither the House nor Senate has passed the FAST Generics Act [32].

#### Strategies for promoting generic drug utilization and development

In order to encourage generic drug development, two strategies have been frequently proposed by researchers and policymakers: implementing initiatives or reforms to increase generic utilization (thereby stimulating the generic economy by inducing demand) and offering aids or incentives for generic drug innovations [9, 33].

Godman and his colleagues proposed a 4 “E” methodology (Education, Engineering, Economics and Enforcement) for developing initiatives to increase generic drug utilization in Europe [8] (Table 2). Initiatives focusing on “Education” are usually programs that influence generic prescribing by disseminating educational materials. Initiatives that focus on organizational interventions (“Engineering”) include agreements on price and volume of existing drugs or disease management programs. Financial incentives (“Economics”) are for increasing generic drug utilization through the use of positive and negative incentives for physicians and patients. Finally, regulatory or law enforcement (“Enforcement”) methods may include mandatory generic substitution laws to which pharmacists must adhere. Some of these initiatives can benefit generic utilization in the U.S., while others may face unique challenges in the U.S. marketplace.

**Table 2:** Strategies for increasing generics development and utilization: the four “E” method [8]

Education	Educational materials: treatment guidance and educational outreach visit
Engineering	Organizational interventions: agreements on price and volume of existing drugs
Economics	Financial incentives: positive and negative incentives for physicians
Enforcement	Regulatory or law enforcement: mandatory generic substitution laws

Indeed, the U.S. healthcare system has mechanisms that promote generic usage. For example, most insurance companies incentivize patients to use generic drugs by requiring less cost-sharing for generic versus branded products. In addition, 14 states currently have mandatory generic substitution laws for pharmacists and the remainder, except Oklahoma, have laws permitting substitution by pharmacists [34]. However, it is unlikely that *federal* laws promoting generic substitution will be enacted due to the organization of the U.S. legal system, and “engineering” initiatives may be challenging given the fragmented nature of the U.S. healthcare system. Evidence is needed about the effects on generic development and utilization of those initiatives already in place, and future research should assess the potential impact of supplemental initiatives such as those involving education of patients and health care providers.

The above activities are promising but all hinge upon the production of new generic products. With more complicated branded products losing their patent protection, bioequivalence methodologies for complex drugs or dosage forms need to be developed. Guidance documents with updated standards or reference documents from the FDA may encourage generic manufacturers to proceed with the development of new products [8, 35]. Regular engagement with OGD may help drug manufacturers contain costs and decrease time-to-market delays. The OGD now has provisions to grant pre-ANDA meetings for complex generics [36].

## Conclusions

We have described in this review select U.S. federal policies, and described the regulatory environment and market forces influencing generic developers. Generic drug developers choose candidate drugs based on the market forces, features of the reference listed drug, and manufacturing capabilities, as well as regulatory restrictions. With suitable policy and regulatory incentives to increase generic utilization or facilitate the generic drug development process, as described, generic manufacturers may be encouraged to develop new generic drugs that will help increase access to medications, improve population health, and contain healthcare spending.

## Abbreviations

ANDA: Abbreviated new drug application
FAST: Fair Access for Safe and Timely
FDA: Food and Drug Administration
FTC: Federal Trade Commission
OGD: Office of Generic Drugs
REMS: Risk evaluation and mitigation strategy

## References

[1] U.S. Department of Health & Human Services, Office of the Assistant Secretary for Planning and Evaluation (2010). ASPE issue brief. Expanding the use of generic drugs.

[2] Anonymous, Generic Pharmaceutical Association (2015). Generic drug savings in the U.S.: Seventh annual edition: 2015.

[3] U.S. Department of Health and Human Services Food and Drug Administration, Center for Drug Evaluation and Research (CDER) (2003). Guidance for industry: bioavailability and bioequivalence studies for orally administered drug products – general considerations.

[4] U.S. Department of Health and Human Services Food and Drug Administration, Center for Drug Evaluation and Research (CDER) (2014). Guidance for industry: bioavailability and bioequivalence studies submitted in NDAs or INDs — general considerations.

[5] U.S. Department of Health and Human Services Food and Drug Administration, Center for Drug Evaluation and Research (CDER) (2013). Guidance for industry: bioequivalence studies with pharmacokinetic endpoints for drugs submitted under an ANDA.

[6] Ashburn TT, Thor KB (2004). Drug repositioning: identifying and developing new uses for existing drugs. Nat Rev Drug Discov.

[7] Barei F, Le Pen C, Simoens S (2013). The generic pharmaceutical industry: moving beyond incremental innovation towards re-innovation. Generics Biosimilar J.

[8] Ding M, Dong S, Eliashberg J, Gopalakrishnan A (2014). Portfolio management in new drug development. Innovation and marketing in the pharmaceutical industry.

[9] Ross MS (2010). Innovation strategies for generic drug companies: moving into supergenerics. IDrugs.

[10] Barei F, Le Pen C, Simoens S (2013). The generic pharmaceutical industry: moving beyond incremental innovation towards re-innovation. Generics Biosimilars Initiat J.

[11] Von Koeckritz K (2012). Generic drug trends—What’s next?. Pharm Times.

[12] McKinsey & Company, Global Generics Interest Group (2013). Generating value in generics: finding the next five years of growth.

[13] Godman B, Shrank W, Andersen M (2010). Comparing policies to enhance prescribing efficiency in Europe through increasing generic utilization: changes seen and global implications. Expert Rev Pharmacoecon Outcomes Res.

[14] Chidambaram A (2013). Global generic pharmaceutical market – Qualitative and quantitative analysis.

[15] U.S. Food and Drug Administration (2016). Product-specific recommendations for generic drug development.

[16] Srinivasan A (2015). Complex generics: maximizing FDA approval potential.

[17] Sario N (2015). Prescription drug market: the world is turning to generics. Market realist.

[18] Lionberger R. Complex Generic Drugs. GPhA Fall Technical Meeting, Bethesda, MD. 2013. Retrieved from http://www.fda.gov/downloads/aboutfda/centersoffices/officeofmedicalproductsandtobacco/cder/ucm374191.pdf.

[19] Bowman D (2015). A quick guide for sourcing biopharmaceutical raw materials. BioProcess international.

[20] Shargel L, Kanfer I, editors. Generic drug product development: solid oral dosage forms. Active Pharmaceutical Ingredients. Chapter 2. New York: CRC Press; 2013. p. 19–21.

[21] American Society of Health-System Pharmacists (2006). ASHP guidelines on handling hazardous drugs. Am J Health Syst Pharm.

[22] Wiske CP, Ogbechie OA, Schulman KA (2015). Options to promote generics markets in the Unites States. JAMA.

[23] Kesselheim AS (2011). An empirical review of major legislation affecting drug development: past experiences, effects, and unintended consequences. Milbank Q.

[24] Federal Trade Commission (2015). Settlement of Cephalon pay for delay case ensures $1.2 billion in Ill-gotten gains relinquished; refunds will go to purchasers affected by anticompetitive tactics.

[25] Federal Trade Commission (2013). Agreements filed with the Federal Trade Commission under the Medicare Prescription Drug, Improvement, and Modernization Act of 2003.

[26] Supreme Court of the United States (2013). Federal Trade Commission v. Actavis, Inc. October term, 2012 No. 12–416.

[27] Hemphill CS, Lemley MA (2011). Earning exclusivity: generic drug incentives and the Hatch-Waxman Act. Antitrust Law J.

[28] Mclean B (2001). Bitter Pill Prozac made Eli Lilly. Then along came a feisty generic maker called Barr Labs. Their battle gives new meaning to the term 'drug war..

[29] Sarpatwari A, Kesselheim AS (2015). Ensuring timely approval of generic drugs. Health Affairs Blog.

[30] Brill A (2014). Lost prescription drug savings from use of REMS programs to delay generic market entry.

[31] The 114^th^ Congress. 1^ST^ Session. H.R.2841. FAST Generics Act of 2015. 2015. Retrieved from https://www.congress.gov/114/bills/hr2841/BILLS-114hr2841ih.pdf.

[32] Anonymous (2015). Fast generics act reintroduced in congress.

[33] Kaló Z, Holtorf AP, Alfonso-Cristancho R, Shen J, Ágh T, Inotai A, Brixner D (2015). Need for multicriteria evaluation of generic drug policies. Value Health.

[34] Survey of Pharmacy Law – 2015 (2014). Mount prospect.

[35] Boehm G, Yao L, Han L, Zheng Q (2013). Development of the generic drug industry in the US after the Hatch-Waxman Act of 1984. Acta Pharm Sin B.

[36] Lionberger R, U.S. Food and Drug Administration (2015). GDUFA regulatory science update. GPhA annual meeting.

